# Human colorectal cancer-derived mesenchymal stem cells promote colorectal cancer progression through IL-6/JAK2/STAT3 signaling

**DOI:** 10.1038/s41419-017-0176-3

**Published:** 2018-01-18

**Authors:** Xiaochao Zhang, Fayong Hu, Geng Li, Guodong Li, Xi Yang, Liang Liu, Rongsheng Zhang, Bixiang Zhang, Yongdong Feng

**Affiliations:** 1 Cancer Research Institute, Tongji Hospital, Tongji Medical College, Huazhong University of Science and Technology, Wuhan, Hubei 430030 China; 2 Hepatic Surgery Centre, Tongji Hospital, Tongji Medical College, Huazhong University of Science and Technology, Wuhan, Hubei 430030 China

## Abstract

Mesenchymal stem cells (MSCs) have been reported to localize in colorectal carcinomas, and participate in the formation of the tumor microenvironment. They have recently been isolated from colorectal cancer tissues, and are implicated in the growth, invasion, and metastasis of cancer cells. However, the roles and detailed mechanisms associated with human colorectal cancer-derived MSCs (CC-MSCs) have not been fully addressed. In this study, we found that CC-MSCs increased the migration and invasion of colorectal cancer cells and promoted the tumorigenesis of colorectal cancer through epithelial-to-mesenchymal transition (EMT) in vitro. We also found that CC-MSCs enhanced the growth and metastasis of colorectal cancer in vivo. Mechanistically, we determined that interleukin-6 (IL-6) was the most highly expressed cytokine in the CC-MSC conditioned medium, and promoted the progression of colorectal cancer cells through IL-6/JAK2/STAT3 signaling, which activated PI3K/AKT signaling. We used anti-IL-6 antibody to target IL-6. Collectively, these results reveal that the IL-6 secreted by CC-MSCs enhances the progression of colorectal cancer cells through IL-6/JAK2/STAT3 signaling, and could provide a novel therapeutic or preventive target.

## Introduction

Mesenchymal stem cells (MSCs) are multi-potent progenitor cells that are present in various normal tissues, including bone marrow, and adipose and liver tissues. They can be induced to differentiate into adipose and bone cells by supplying them with the appropriate culture medium^1–4^. Apart from these normal tissues, MSCs have recently been isolated from various tumor tissues, and take part in the formation of the tumor stroma. For example, multi-potent MSCs have been isolated from lipomas, gastric tumors, bone sarcomas, and the tumor stroma of a mouse model^5–8^. Moreover, the MSCs derived from the tumor tissues mentioned above have similar phenotypes: a long, spindle-shaped morphology; parallel surface markers; and the ability to differentiate into adipose cells, chondrocytes, and bone cells.

Consequently, MSCs contribute to the regeneration of various tissues^9^. They are recruited to inflamed or damaged tissues by local endocrine signals, resulting in the formation of fibrous scars^10,11^. As with scar formation and wound healing, the growth of tumor tissues is associated with abundant matrix-remodeling proteins, cytokines, and growth factors, which explains why tumors are associated with wounds that never heal^12^. This indicates that growing tumors recruit MSCs by secreting numerous endocrine and paracrine hormones. However, the interactions between MSCs and cancer are obscure. Recently it has been reported that the injection of MSCs and tumor cells promotes tumor growth and metastasis^13–21^. Researchers have reported that MSCs are involved in tumor invasion and angiogenesis^13–15,21^, immunosuppression^16,17^, and apoptosis suppression^19^. Shinagawa et al.^22^ have reported the importance of tumor and MSC interactions in the growth and metastasis of colon cancer. Tumor evolution is stimulated by direct cell–cell contact or by the paracrine secretion by MSCs of cytokines and growth factors such as epidermal growth factor (EGF), interleukin-6 (IL-6), vascular epidermal growth factor (VEGF), insulin-like growth factor 1 (IGF-1), or transforming growth factor beta (TGF-β)^23–28^. Several cytokines, especially IL-6 and IL-8, may have a significant influence on cancer progression.

IL-6 is a cytokine; it accompanies inflammation, and is involved in the progression of cancers, including colorectal cancer. The authors of one study reported a higher level of serum IL-6 in patients suffering from colorectal cancer than in a healthy control group^29^. Moreover, IL-6 can act as a paracrine cytokine to promote the proliferation of colorectal cancer cells^30^. IL-6 can activate several signaling pathways, including STAT, ERK/MAPK, and PI3K/AKT. It has been reported that IL-6 promotes the proliferation and invasion of colorectal cancer cells through Ras/MAPK and PI3K/AKT signaling^31^.

In the present study, we isolated colorectal cancer-derived MSCs (CC-MSCs) from primary human colorectal cancer tissues, and identified their phenotype. We then investigated their effect on the growth and metastasis of colorectal cancer compared with a control. We also investigated the mechanisms underlying the tumor-promoting effect of CC-MSCs.

## Results

### Isolation, morphology, and differentiation capability of human colorectal cancer-derived MSCs

**Fig. 1:**
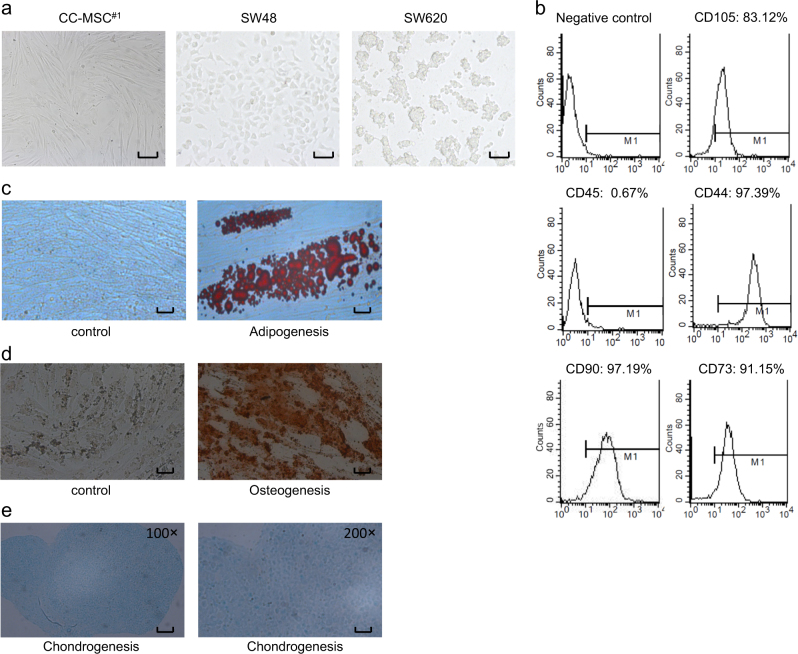
The characterizations of human colorectal cancer-derived mesenchymal stem cells (CC-MSCs). **a** Morphology of CC-MSCs, SW620 colorectal cancer cells, and SW48 colorectal cancer cells (magnification, ×100; scale bar: 250 μm). The CC-MSCs were fibroblastic in shape, but the SW620 and SW48 cancer cells were round. **b** Flow cytometric analysis of CC-MSCs. The CC-MSCs were positive for CD105, CD90, CD73, and CD44, and negative for CD45. **c** CC-MSCs cultivated in control medium or adipogenic differentiation medium were stained with Oil Red O. The red lipid droplets indicate adipogenic differentiation (magnification, ×100; scale bar: 250 μm). **d** CC-MSCs cultivated in control medium or osteogenic differentiation medium were stained with alizarin red (magnification, ×100; scale bar: 250 μm). **e** CC-MSCs cultivated in chondrogenic differentiation medium were stained with alcian blue (left panel, magnification, ×100; scale bar: 250 μm; right panel, magnification, ×200; scale bar: 125 μm)

We cultured MSC-like cells with typical long-spindle morphology from human colorectal cancer tissues under MSC nutritional conditions (Fig. 1a). To culture the CC-MSCs, we obtained five fresh human colorectal cancer samples. As with human bone marrow MSCs, the CC-MSCs were dispersed and shaped like long spindles. Flow cytometric analysis revealed that the CC-MSCs were positive for CD105, CD90, CD73, and CD44, and negative for CD45 (Fig. 1b). To confirm the differentiation ability of the CC-MSCs, we cultivated them in adipogenic differentiation media, osteogenic differentiation medium and chondrogenic differentiation medium. CC-MSCs could differentiate into adipocytes, osteocytes and chondrocytes which were verified by Oil Red O, alizarin red and alcian blue staining (Fig. 1c, d, e).

### CC-MSCs significantly enhanced the migration and invasion of colorectal cancer cells, and mediated epithelial-to-mesenchymal transition (EMT) in colorectal cancer

**Fig. 2:**
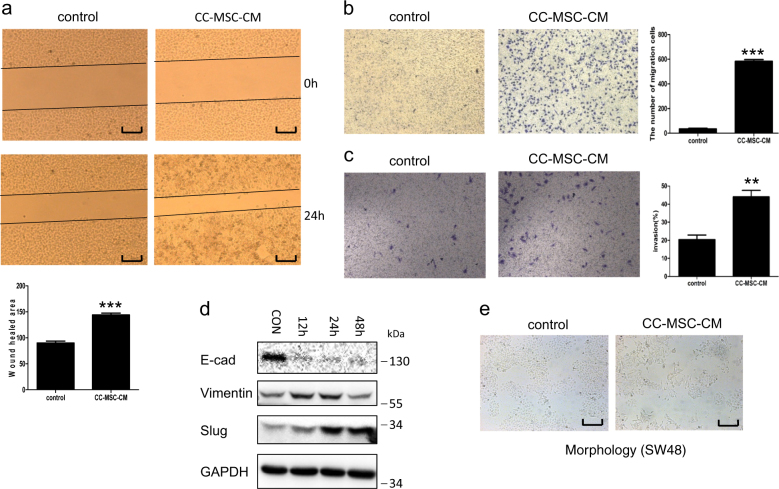
Human colorectal cancer-derived mesenchymal stem cells promote the migration and invasion of SW48 colorectal cancer in vitro, and mediate epithelial-to-mesenchymal transition (EMT) in colorectal cancer. **a** Wound-healing closure assays of SW48 colorectal cancer cells were performed in the presence or absence of CC-MSC conditioned medium (CC-MSC-CM) (magnification, ×50; scale bar: 500 μm). **b** Chemotaxis assays of SW48 cells with CC-MSCs-CM in the lower chamber (×100). **c** Invasion assays of SW48 colorectal cancer cells were also carried out in the presence or absence of CC-MSCs-CM (×100). The addition of the CC-MSC-CM enhanced both the migration and invasion of the SW48 cells. *P < 0.05 compared with mock treatment. **d** SW48 cells were cultivated with CC-MSC-CM for different times, and several markers associated with the EMT process, such as E-cadherin, vimentin, and SLUG, were detected by western blotting analysis. F12 served as the control. **e** SW48 cells in the presence or absence of CC-MSC-CM were photographed using a phase contrast microscope. A mesenchymal phenotype in SW48 cells was viewed in the presence of CC-MSC-CM and compared with the control group (magnification, ×50; scale bar: 500 μm)

To determine whether CC-MSCs have a significant impact on the migration of colorectal cancer cells, we examined SW48 and SW480 cells in the presence or the absence of CC-MSC-CM. The results revealed that CC-MSC-CM enhanced migration in the SW48 and SW480 cells to a significantly greater degree than in the controls (Fig. 2a, b, Supplementary Figure 1a and b). Similarly, to determine whether CC-MSC-CM enhanced the invasion of colorectal cancer cells, we examined the invasive ability of SW48 and SW480 in the presence or absence of CC-MSC-CM. We found that CC-MSC-CM greatly promoted the invasion of SW48 and SW480 (Fig. 2c, Supplementary Figure 1c). The results suggest that soluble factors secreted by the CC-MSCs were responsible for the effects. EMT is a significant step in invasion and metastasis progression^32^. We found that SW48 cells cultivated with CC-MSCs-CM expressed reduced levels of E-cadherin, the epithelial marker, but increased levels of vimentin, the mesenchymal marker. SLUG is the key transcription factor in EMT. We showed that the expression of SLUG was enhanced by CC-MSCs (Fig. 2d). Moreover, SW620 cells cultivated with CC-MSCs-CM produced similar results (Supplementary Figure 1d). To confirm these findings at the mRNA level, molecular analyses with quantitative PCR (qPCR) assays were conducted on SW48 cells (Supplementary Figure 1e). We also showed that in the presence of CC-MSCs-CM, the SW48 cells had a mesenchymal phenotype compared with the control group (Fig. 2e).

### CC-MSCs had a positive effect on colorectal cancer cell stemness, and promoted angiogenesis in vitro

**Fig. 3:**
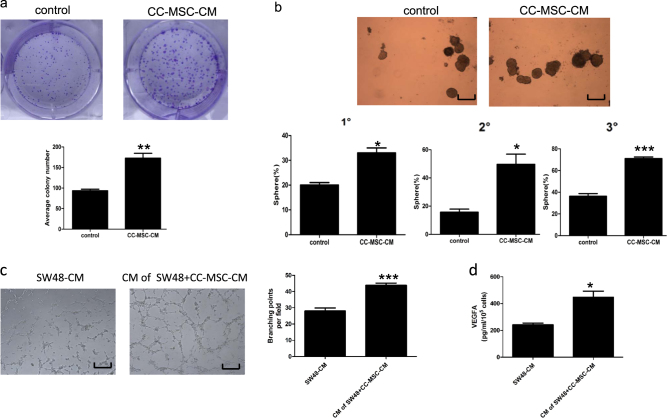
Human colon cancer-derived mesenchymal stem cells promote the generation of cancer stem cells and enhance angiogenesis in vitro. **a** SW48 cells treated with CC-MSC-CM were assayed for clonogenicity in adherent cultures. Separated clones were counted and plotted. **b** Effect of CC-MSC-CM on serial sphere-formation assays of SW620. Tumor spheres were generated in suspension culture for 2 weeks using Ultra-Low Attachment plates (magnification, ×100; scale bar: 250 μm). *P < 0.05 compared with mock treatment. **c** Human umbilical vein endothelial cells (HUVECs) were cultivated in CM from control SW48 cells and SW48 cells pretreated with CC-MSC-CM for 24 h. The cells’ tube formation ability was quantified by counting the branching points per field (magnification, ×100; scale bar: 250 μm). ***P < 0.001. **d** ELISA was used to examine the VEGFA level in the CM obtained in Fig. 3c. All experiments were carried out in triplicate. *P < 0.05

To determine whether CC-MSCs promote colony formation in cancer cell lines, we seeded 1000 SW48 and SW480 cells in the colony-forming assay. MSC-induced clones were larger and more prevalent in the SW48 and SW480 cells than in the control groups (Fig. 3a, Supplementary Figure 2a). The cultivation of SW620 with CC-MSC-CM induced an obvious enhancement of cancer cell stemness compared with cultivation in F12, as demonstrated by the sphere-forming assays. For the serial sphere-formation assays, the first-generation spheres were harvested and the sphere-forming assays were performed continually. This course was repeated for up to three generations. The serial sphere-formation assays also indicated that the CC-MSCs significantly promoted the generation of cancer stem cell (Fig. 3b). We also determined whether CC-MSCs promote angiogenesis by carrying out a tube formation assay in vitro. We determined that the CM obtained from the SW48 cells pretreated with CC-MSC-CM enhanced tube formation in HUVECs compared with the CM from the control SW48 cells (Fig. 3c). Furthermore, we conducted ELISA and real-time PCR to detect VEGFA expression levels in both groups. The VEGFA level in the CM obtained from SW48 cells pretreated with CC-MSC-CM was much higher than that in the CM from the control SW48 cells (Fig. 3d, Supplementary Figure 3a).

### IL-6 secretion was noticeably high in the conditioned medium of the CC-MSCs, and maintained the proliferation, migration and invasion of colorectal cancer cells

**Fig. 4:**
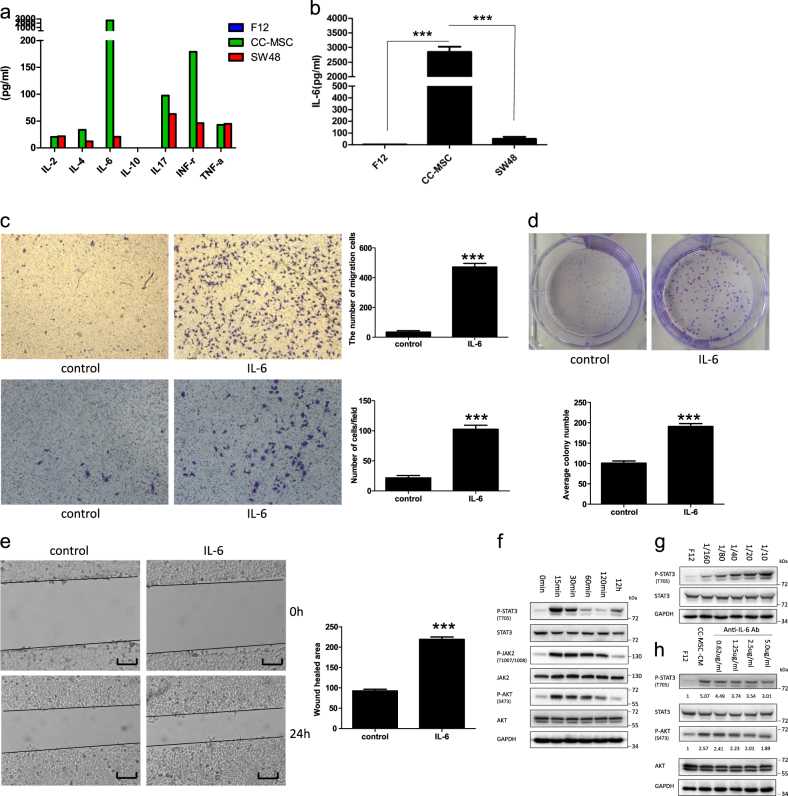
IL-6 secreted by CC-MSCs enhances the proliferation, migration and invasion of colorectal cancer cells through IL-6/JAK2/STAT3 signaling. **a** The levels of various factors in the cell-free culture supernatants were measured using Bio-Plex cytokine arrays. **b** Quantitative analysis of IL-6 levels using enzyme-linked immunosorbent assay (ELISA). The conditioned media from the cultured CC-MSCs and SW48 cells were collected to detect the levels of IL-6, and representative results from one of the three independent experiments are presented. **c** Transwell migration (top) and invasion (bottom) assay of SW48 cells with or without 10 ng/mL recombinant IL-6 treatment (×100). **d** Colony-formation assay of SW48 cells treated with or without 10 ng/mL recombinant IL-6. **e** Wound-healing assay of SW48 cells in the presence or absence of recombinant IL-6 (magnification, ×50; scale bar: 500 μm). ***P < 0.001. **f** SW48 cells were cultured with CC-MSC-CM for different times. Protein expression was determined by western blotting and representative results from one of the three independent experiments are presented. **g** SW48 cells were cultured with different dilution ratios of CC-MSC-CM for 30 min. Protein expression was determined by western blotting and representative results from one of the three independent experiments are presented. **h** SW48 cells were cultured for 30 min in F12 or CC-MSC-CM, which had been pre-incubated with different concentrations of anti-IL-6 antibody for 2 h, and representative results from one of the three independent experiments are presented

Because of the enhanced effect of CC-MSCs-CM on the progression of colorectal cancer cells, we used a cytokine array to examine the cytokine secretion profiles of the CC-MSCs. We cultivated CC-MSCs in serum-starved F12 for 24 h. We then harvested and filtered the conditioned media, and determined the cytokine levels. Of the cytokines examined, IL-6 and INF-γ were noticeably abundant in the CC-MSCs, but not in the SW48 cells (Fig. 4a). However, the IL-6 level increased over a larger range than any other cytokines. We determined the levels of IL-6 in CC-MSCs-CM and SW48-CM using ELISA (Fig. 4b). To further confirm the role of IL-6 in the progression of colorectal cancer cells, recombinant IL-6 protein was used. Treatment with IL-6 enhanced cancer cell migration, invasive (Fig. 4c, Supplementary Figure 4a) and proliferation (Fig. 4d, Supplementary Figure 4b). Also, the size of the wound in the wound-healing assay narrowed under treatment with recombinant IL-6 (Fig. 4e, Supplementary Figure 4c). Taken together, these results demonstrated that IL-6, secreted from CC-MSCs, contributed to colorectal cancer progression.

### CC-MSCs conditioned medium promoted the activation of STAT3 and PI3K/AKT through IL-6/JAK2/STAT3 signaling in colorectal cancer cells

We examined protein levels by western blotting to investigate the mechanism underlying the promotion of progression in colorectal cancer cells. Western blotting analysis revealed that CC-MSC-CM resulted in increased phosphorylation of JAK2, STAT3, and AKT in colorectal cancer cells; all three were activated by stimulation with IL-6 (Fig. 4f, Supplementary Figure 4d). Within approximately 15–30 min, JAK2, STAT3, and AKT were activated in SW48 cells treated with CC-MSC-CM, and STAT3 activation took place in a dose-dependent manner (Fig. 4g). After the application of anti-IL-6 antibody, STAT3 and AKT activation was partly inhibited (Fig. 4h, Supplementary Figure 4e). These data indicated that the activation of IL-6/JAK2/STAT3 signaling was required for the activation of AKT, which resulted in the colorectal cancer-promoting effect of CC-MSCs.

### Anti-IL-6 antibody and an inhibitor of STAT3 attenuated the colorectal cancer-promoting effect of the CC-MSCs

**Fig. 5:**
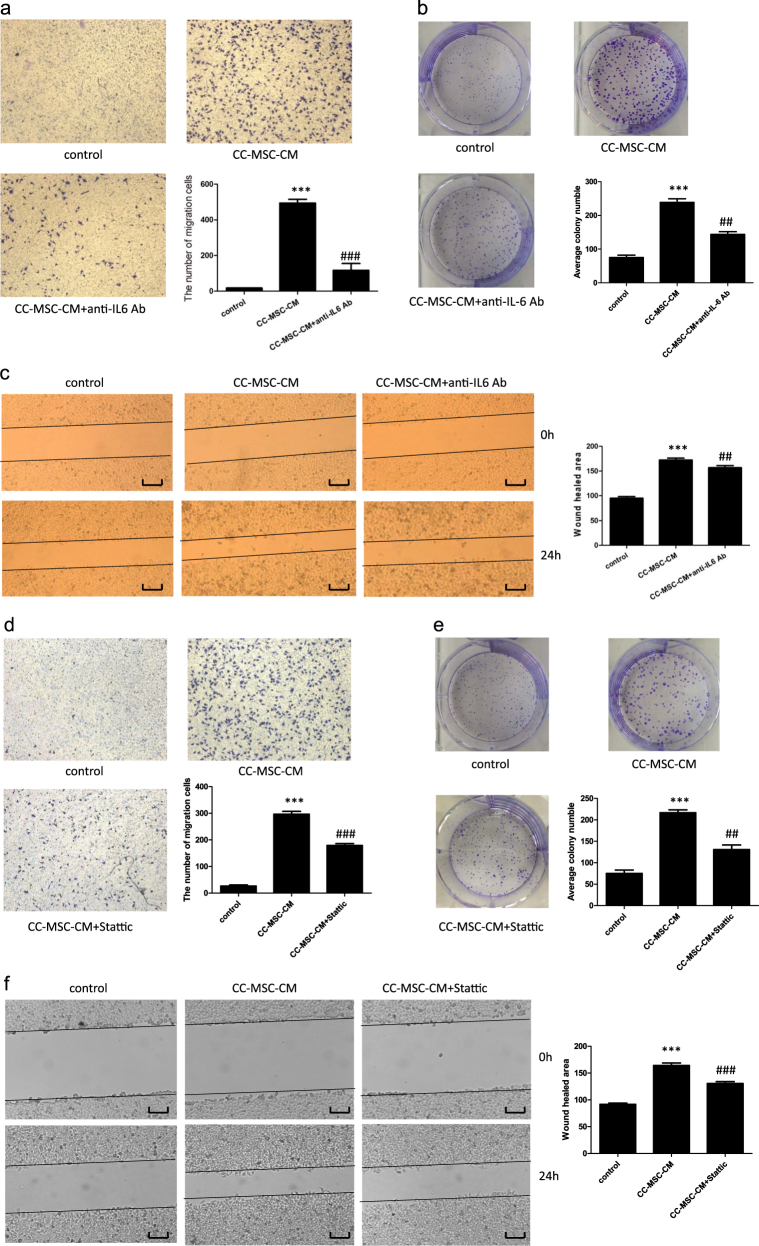
Tumor-promoting effect of CC-MSCs on SW48 cells is reduced by the addition of anti-IL-6 antibody and the inhibitor of STAT3. **a** Transwell migration assay of SW48 cells exposed to CC-MSC-CM with or without anti-IL-6 antibody treatment (×100). **b** Colony-formation assay of SW48 cells treated with CC-MSC-CM in the presence or absence of anti-IL-6 antibody. **c** Wound-healing assay of SW48 cells exposed to CC-MSC-CM was performed in the presence or absence of anti-IL-6 antibody (magnification, ×50; scale bar: 500 μm). **d** Transwell migration assay of SW48 cells exposed to CC-MSC-CM with or without the STAT3 inhibitor (Stattic) treatment (×100). **e** Colony-formation assay of SW48 cells treated with CC-MSC-CM in the presence or absence of the STAT3 inhibitor (Stattic). **f** Wound-healing assay of SW48 cells exposed to CC-MSC-CM was performed in the presence or absence of the STAT3 inhibitor (Stattic) (magnification, 50X; scale bar: 500 μm). *P < 0.05, **P < 0.01, ***P < 0.001: compared with the control group; ^#^*P* < 0.05, ^##^*P* < 0.01, ^###^*P* < 0.001: compared with the CC-MSC-CM-treated group

Because IL-6 was known to play a significant role in tumor progression and extremely high levels of IL-6 were detected in the study described above, we postulated that CC-MSCs promoted IL-6-mediated tumor migration. Transwell assays revealed that simultaneous treatment with CC-MSCs-CM and anti-IL-6 antibody caused a significant reduction in the migration of SW48 cells compared with the results for the CC-MSC-CM-only group (Fig. 5a, Supplementary Figure 5a). The colony number, which was enhanced by CC-MSC-CM in the colony-formation assay, was drastically reduced by anti-IL-6 antibody (Fig. 5b, Supplementary Figure 5b). Furthermore, the size of the wound in the wound-healing assay expanded under simultaneous treatment with CC-MSC-CM and anti-IL-6 antibody compared with the CC-MSC-CM-only group (Fig. 5c, Supplementary Figure 5c). To test whether activation of STAT3 was required for the colorectal cancer-promoting effect of the CC-MSCs, Stattic, a specific chemical inhibitor of STAT3, was used. Inhibition of STAT3 significantly minimized the effect of CC-MSCs on the migration of SW48 cells (Fig. 5d, Supplementary Figure 5d). In addition, we found that treating SW48 cells with CC-MSC conditioned media increased the colony number compared with control media, which was diminished by Stattic treatment (Fig. 5e, Supplementary Figure 5e). Moreover, Stattic reduced the increased wound healed area induced by CC-MSC-CM (Fig. 5f, Supplementary Figure 5f). Taken together, these data showed that the effect of the CC-MSCs on colorectal cancer progression was dependent on IL-6 and STAT3 activation.

### CC-MSCs enhanced colorectal cancer cell growth and metastasis in vivo

**Fig. 6:**
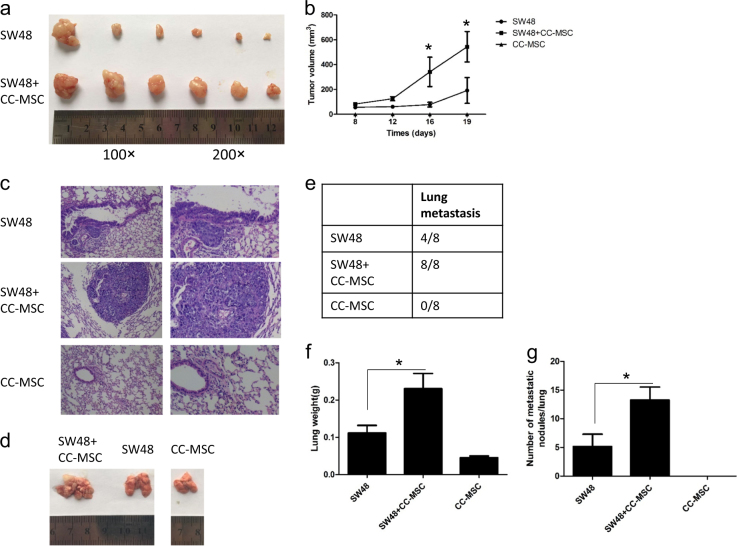
Human colorectal cancer-derived mesenchymal stem cells enhance the growth and metastasis of colorectal cancer in vivo. **a** Subcutaneous tumors from SW48 cells injected into the flank of nude mice with or without CC-MSCs (1:1). **b** Volume of subcutaneous tumors in mice with SW48 cells with or without CC-MSC transplantation, or CC-MSCs alone (*n* = 6). **c** Representative hematoxylin and eosin staining and image **d** of lung tissue sections from BALB/c (nu/nu) mice from each group killed at 8 weeks. **e** Incidence of lung metastasis in the different groups of nude mice (*n* = 8). **f** Weight of lung tissue in each group. **g** Number of lung metastatic foci in each group (*n* = 8). *P < 0.05 compared with the SW48 alone group

We used a xenograft model to examine the role of CC-MSCs in colorectal cancer in vivo. SW48 cells alone, CC-MSCs alone, or SW48 cells mixed with CC-MSCs were injected into the flanks of female nude mice. The tumors produced by co-injection of SW48 cells and CC-MSCs were significantly larger than those produced by SW48 cells alone (Fig. 6a). No tumors were formed following the injection of CC-MSCs alone (Fig. 6b). To confirm the effect of CC-MSCs on tumor metastasis in vivo, we injected SW48 cells alone, CC-MSCs alone, or SW48 cells mixed with CC-MSCs into nude mice via the tail vein. The mice were sacrificed 8 weeks later (*n* = 8). Compared with SW48 cells alone, co-injection of SW48 cells and CC-MSCs (1:1) increased the number of tumor nodules (Fig. 6d, f, g), and the group with larger metastatic nodules in the lung tissue was detected by hematoxylin and eosin staining (Fig. 6c). The metastatic potential of the SW48 cells was significantly enhanced by the presence of CC-MSCs. The injection of CC-MSCs alone did not result in the formation of metastatic tumors in the lungs (Fig. 6e). These results demonstrate that CC-MSCs enhance the growth and metastasis of colorectal cancer in vivo.

## Discussion

Solid tumors grow in a complicated microenvironment that comprises cancer-associated fibroblasts, macrophages, MSCs, neutrophils, and extracellular matrix. The tumor and surrounding stroma interact with each other, resulting in cancer progression. The authors of a recent study have reported that bone marrow-derived MSCs (BM-MSCs) are present in the tumor microenvironment and participate in the promotion of tumor growth^33^. BM-MSCs are recruited to the tumor stroma and integrated into the stroma microenvironment^34,35^. Despite extensive research confirming the correlation between various normal tissue-derived MSCs and tumors, tumor-derived MSCs and their roles in tumor progression deserve further investigation.

In the current study, we isolated MSCs from human colorectal cancer tissues and confirmed that they had multi-lineage differentiation potential; the surface markers and morphology of the isolated CC-MSCs had the usual features of MSCs. We confirmed that the CC-MSCs enhanced the migration and stemness of colorectal cancer cells in vitro, and promoted the growth and metastasis of colorectal cancer in vivo. We also attempted to determine the potential mechanism associated with the pro-tumor effects of CC-MSCs.

To begin with, we determined that CC-MSCs have an influence on the progression of colorectal cancer cells. The results showed that the colorectal cancer cells were encouraged to migrate more towards CC-MSCs-CM than the control, which demonstrated that CC-MSCs have the potential to enhance colorectal cancer metastasis. We also demonstrated that CC-MSCs enhance the growth and metastasis of colorectal cancer in vivo. Similar to our findings, Li et al.^36^ reported that gastric cancer-derived MSCs have a more potent effect on the promotion of tumor migration than BM-MSCs or MSCs from adjacent non-cancerous tissue. Another study demonstrated that BM-MSCs promote breast cancer metastasis^21^. Moreover, we determined the effect of CC-MSCs on cancer cell stemness using an ultra-low attachment plate. CC-MSCs enhanced the stemness of colorectal cancer cells more than the control, which suggests an important role of CC-MSCs in the promotion of tumorigenesis in colorectal cancer. Furthermore, CC-MSCs have a positive effect on angiogenesis.

Next, because CC-MSCs can enhance the migration and invasion of colorectal cancer cells, we speculated whether CC-MSCs can promote ETM transition. Consequently, we determined that colorectal cancer cells incubated in CC-MSCs-CM had elevated levels of vimentin and SLUG, and reduced levels of E-cadherin, which illustrated that CC-MSCs promote colorectal cancer metastasis via inducing ETM transition. We also investigated the mechanism underlying the pro-tumor effect of CC-MSCs. According to a report by Rokavec et al., IL-6 enhances EMT-mediated colorectal cancer invasion and metastasis^37^. Huynh et al.^38^ reported that IL-6 levels are elevated in colorectal cancer, and the major source of IL-6 is CD90+ stromal cells, which proves the presence of cancer stem-like cells and inflammation in colorectal cancer. However, the mechanism has not been decisively demonstrated in colorectal cancer. In the present study, we detected higher levels of IL-6 expression in the CC-MSCs than in the control, which suggested that IL-6 probably plays a significant part in the invasion and metastasis of colorectal cancer. Subsequently, we determined the effect of colorectal cancer-derived MSC-CM on colorectal cancer cells in the presence and in the absence of IL-6 antibody. We observed that anti-IL-6 antibody markedly attenuated the migration induced by CC-MSC-CM, which demonstrated that IL-6 has a significant role in the promotion of metastasis in colorectal cancer.

We also illustrated that the JAK2 and STAT3 activation play a significant role in the promotion of metastasis in colorectal cancer. JAK2 and STAT3 activation have an important effect on metastasis in colorectal cancer. STAT3 is activated by IL-6 in the tumor microenvironment^39^. Tsai et al. demonstrated that JAK2 and STAT3 activation by IL-6 is important for tumor progression in colon cancer cells^40^. Bartolomé et al. reported that PI3K/AKT activation promotes metastasis in colon cancer^41^. In the present study, we demonstrated that CC-MSCs increase the activation of AKT in colorectal cancer cells, and this effect was shown to be partly attenuated in the presence of IL-6 antibody, implying that CC-MSCs-secreted IL-6 promotes the migration and invasion of colorectal cancer cells through the signal pathways associated with tumor metastasis. Therefore, these results indicate that the enhancing effect of CC-MSCs on colorectal cancer cell migration and invasion is due to the extremely high levels of IL-6 that they secrete. Ameliorating the interaction between CC-MSCs and colorectal cancer cells using anti-IL-6 antibodies may offer a novel therapeutic or preventive treatment.

However, our experiment had several limitations; we only used three colorectal cancer cell lines, and an in-depth investigation to determine the mechanisms in other tumor models is necessary. Moreover, STAT3 activation dropped after 120 min of CC-MSC-CM medium treatment, but increased again after 12 h (Fig. 4f). The phenomenon was strange. A previous study had reported that STAT3 activation was as early as 30 min of IL-6 treatment and in a dose-dependent manner, but dropped after 90 min^40^. Another study had also reported that robust elevated phosphorylation of pSTAT3^Y705^ was detected after 5 min of IL-6 treatment of primary hepatocytes, but STAT3 activation dropped after 120 min^42^. These results were consistent with ours, so we consider that the increase of STAT3 activation after 12 h might not be related to IL-6 treatment. The phenomenon might be associated with autonomous slight STAT3 activation in colorectal cancer cells, which needed to be further investigated.

In brief, our results indicate that CC-MSCs enhance colorectal cancer progression through the activation of IL-6/JAK2/STAT3 signaling.

## Materials and methods

### Ethics statement

Colorectal cancer tissues were obtained from patients who had undergone surgery at the Tongji Hospital, Huazhong University of Science and Technology. Written informed consent was obtained from all participants, and all procedures were authorized by the Ethical Committee of Tongji Hospital.

### Isolation of MSCs from colorectal cancer tissues and subsequent primary culture

Fresh colorectal adenocarcinoma tissues were obtained from five patients who had undergone surgery. The specimens were initially soaked in 95% ethanol to protect them from contamination, then washed in phosphate-buffered saline with 1% penicillin/streptomycin. We used scissors to cut the specimens into sections, which were then digested with collagenase IV (Invitrogen, CA, USA) for 3 h at 37 °C. After digestion, we used phosphate-buffered saline (PBS) to wash the tissues, which we then passed through a 70 µm mesh (BD Falcon, CA, USA). We centrifuged the filtrate and cultivated the cells in red blood cell lysis buffer to eliminate red blood cells, then washed the remaining cells three times with PBS. The cells were then plated in Dulbecco’s modified Eagle’s medium (DMEM) with low glucose content containing 10% fetal bovine serum (FBS; Gibco, USA) and 1% penicillin/streptomycin. The culture medium was renewed every two days after seeding. The cells were digested with 0.25% trypsin and expanded when cell confluence reached 70%. The human CC-MSCs were ready for use in subsequent experiments after the fourth passage.

### Cell lines and reagents

We obtained human colorectal cancer cells (SW480, SW620, and SW48) from the American Type Culture Collection. The SW480, SW620, and SW48 cells were incubated in DMEM with high glucose content (Invitrogen, CA, USA) containing 10% FBS and 1% penicillin/streptomycin at 37 °C in 5% CO_2_ and 95% air. Stattic (MedChem Express, NJ, USA), a specific chemical inhibitor of STAT3; Anti-IL-6 antibody (Biolegend, CA, USA), a neutralizing antibody for IL-6.

### Adipogenic and osteogenic differentiation

The CC-MSCs in growth medium were seeded at a concentration of 2 × 10^4^ cells/cm^2^ on a six-well plate. Adipogenic differentiation medium A comprising adipogenic differentiation basal medium A, adipogenic differentiation medium fetal bovine serum, penicillin-streptomycin, glutamine, insulin, IBMX, rosiglitazone, dexamethasone (Cyagen Biosciences, CA, USA), was added until the cells were 100% confluent or post confluent. After 3 days, we changed the medium to adipogenic differentiation medium B comprising adipogenic differentiation basal medium B, adipogenic differentiation medium fetal bovine serum, penicillin-streptomycin, glutamine, and insulin (Cyagen Biosciences, CA, USA), then changed it back to adipogenic differentiation medium A after a further 24 h. After cultivation for approximately 3 weeks, we stained the CC-MSCs with Oil Red O (Cyagen Biosciences, CA, USA) to confirm the presence of lipid droplets. CC-MSCs were cultivated in osteogenic differentiation medium, containing osteogenic differentiation basal medium, osteogenic differentiation fetal bovine serum, penicillin-streptomycin, glutamine, ascorbate, β-glycerophosphate, dexamethasone (Cyagen Biosciences, CA, USA). After cultivation for ~4 weeks, we stained the CC-MSCs with alizarin red (Cyagen Biosciences, CA, USA). CC-MSCs were cultivated in Chondrogenic differentiation medium, containing chondrogenic differentiation basal medium, dexamethasone, ascorbate, ITS + Supplement, sodium pyruvate, proline, TGF-β3 (Cyagen Biosciences, CA, USA). After cultivation for approximately 4 weeks, we stained the CC-MSCs with Alcian blue (Cyagen Biosciences, CA, USA).

### Immunophenotyping of CC-MSCs

We carried out flow cytometry to confirm the surface markers of the CC-MSCs at the third passage. The CC-MSCs (1 × 10^6^) were stained with anti-CD105, anti-CD44, anti-CD73, anti-CD90 and anti-CD45 antibodies (antibodies were purchased from BD Bioscience, Heidelberg, Germany). We then analyzed the cells using flow cytometry. APC- or PE-IgG1 (BD Bioscience, Heidelberg, Germany) was used as the control.

### Collecting CC-MSCs conditioned medium

We seeded 2 × 10^6^ CC-MSCs onto a 10-cm plate and cultivated them for 36 h with the complete medium. After 36 h, we replaced the medium with 5 ml of fresh F12/DMEM without serum and cultured the cells for a further 24 h. We collected the CC-MSCs culture medium, filtered it, and stored it at −40 °C.

### Migration, invasion, wound-healing closure, and clonogenicity assay

We investigated the migration and invasion of SW480 and SW48 cells using Transwell chambers (Corning, MA, USA) in a 24-well plate containing 8-µm pores. Serum-free tumor cells in F12/DMEM were placed in the upper chamber and F12/DMEM containing 20% FBS was placed in the lower chamber. We used an upper chamber that had been pretreated with Matrigel coating (2 mg/ml) for the invasion assay, and an untreated upper chamber for the migration assay. After culturing for 24 h, the cells that had migrated towards or invaded the lower chamber were fixed with 4% paraformaldehyde for 10 min, stained with crystal violet, and counted by bright-field microscopy. We grew tumor cells to 95% confluence in DMEM without FBS overnight for the wound-healing closure assay. We scratched the monolayer of cells using a 10 µl pipette tip. The cells were cultivated in the presence or absence of serum-free CC-MSCs-conditioned medium (CC-MSCs-CM) for 24 h, with F12/DMEM as the control. For the clonogenicity assay, 1 × 10^3^ or 5 × 10^2^ SW48 cells were seeded on a 6-well plate in complete F12/DMEM or CC-MSCs-CM with 10% FBS. The cells were cultured for ~14 days. We then counted the separate adherent clones.

### Sphere-formation assay

The fundamental processes of the sphere-formation assay have been described previously^18,40^. The tumor cells were seeded in sphere-forming medium comprising DMEM/F12 (Invitrogen, CA, USA) containing 1 × B27 serum substitute (Invitrogen, CA, USA), 20 ng/ml basic fibroblast growth factor, and 20 ng/ml human recombinant epidermal growth factor (Sigma, St. Louis, USA). We seeded SW620 cells at a concentration of 200 cells/well on a 24-well ultra-low attachment plate (Corning, MA, USA). For the serial sphere-formation experiments, the first-generation spheres were harvested, digested using 0.025% trypsin/ethylene diamine tetraacetic acid (EDTA), passed through a 40-μm mesh, and re-plated in the same way. The process was repeated for up to three generations.

### Western blotting

Western blotting was carried out according to the standard protocols described previously. We used primary antibodies raised against GAPDH (Santa Cruz Biotechnology, CA, USA), E-cadherin, SLUG, vimentin, STAT3, and phospho-STAT3 (Y705) (Cell Signaling Technology, MA, USA). Goat anti-mouse and anti-rabbit antibodies conjugated with horseradish peroxidase were used as secondary antibodies (Jackson ImmunoResearch, PA, USA), and we detected the blots using enhanced chemiluminescence (ECL) (Dura, Pierce, NJ, USA).

### RNA extraction and real-time polymerase chain reaction (PCR) assays

RNA was extracted with TRIzol reagent (Invitrogen, CA, USA), and the isolated RNA was reverse-transcribed into complementary DNA (cDNA) using a Superscript Reverse Transcriptase Kit (Transgene, France) according to the standard instructions. qPCR was carried out on an ABI7300 real-time PCR system using a Super SYBR Green kit (Transgene, France). We used the following specific primer pairs to quantify the expression of the genes that encode the proteins indicated: VEGFA (5′-AGGGCAGAATCATCACGAAGT-3′ and 5′-AGGGTCTCGATTGGATGGCA-3′); E-cadherin (5′- ATTTTTCCCTCGACACCCGAT-3′ and 5′-TCCCAGGCGTAGACCAAGA-3′); Vimentin (5′-AGTCCACTGAGTACCGGAGAC-3′ and 5′-CATTTCACGCATCTGGCGTTC-3′); Slug (5′-CGAACTGGACACACATACAGTG-3′ and 5′-CTGAGGATCTCTGGTTGTGGT-3′); GAPDH (5′GAGAGACCCTCACTGCTG-3′ and 5′-GATGGTACATGACAAGGTGC-3′); β-actin (5′-CATGTACGTTGCTATCCAGGC-3′ and 5′-CTCCTTAATGTCACGCACGAT-3′); Tubulin (5′-AAGATCCGAGAAGAATACCCTGA-3′ and 5′-CTACCAACTGATGGACGGAGA-3′). Gene amplification was carried out according to the standard instructions: 95˚°C for 10 min; and 40 cycles of 95 °C for 15 s and 60 °C for 1 min. Relative quantification was accomplished by normalizing the expression levels of the other genes to *GAPDH*, with all tests carried out in duplicate.

### Quantitation of IL-6 and VEGFA by enzyme-linked immunosorbent assay (ELISA)

We collected the conditioned medium from the CC-MSCs, and used a human IL-6 ELISA kit (eBioscience, CA, USA) to determine the level of IL-6 expression in the CC-MSCs. All tests were performed in duplicate. The plate was read at a wavelength of 450 nm. The IL-6 concentration (pg/ml) was defined by making a standard curve with recombinant IL-6. F12 without supplements served as the control. VEGFA expression was investigated in a similar way using a human VEGFA ELISA kit (eBioscience, CA, USA).

### Tube formation assay

We investigated in vitro angiogenesis using a tube formation assay. Growth factor-reduced Matrigel (Corning, MA, USA) was coated on a 96-well culture plate (100 µl/well), and incubated for 30 min at 37 °C to form a gel. Human umbilical vein endothelial cells (HUVECs; 2 × 10^4^ cells) were seeded into each well and cultivated in the appropriate conditioned medium for 24 h. Tubes were detected using a light microscope every 4 h by examining overall branch points.

### Animal assays

Animal assays were performed according to Wuhan Medical Experimental Animal Care Guidelines. We bred female BALB/c (nu/nu) mice under specific pathogen-free (SPF) conditions and used them until they were 4–5 weeks old. For the tumor growth assay, the mice were divided into three randomized groups (*n* = 6 per group), and 1 × 10^6^ CC-MSCs, or 1 × 10^6^ SW48 cells, or both 1 × 10^6^ SW48 cells and 1 × 10^6^ CC-MSCs in 200 μl were subcutaneously injected into the flank of each mouse. After 8 days, we began measuring the tumor size every 3 days using digital vernier calipers, and calculated the tumor volume according to the following formula: volume = 1/2 × (width^2^ × length). The mice were sacrificed after 19 days, and the tumors were collected and visually examined. For the in vivo lung metastasis experiment, cells were injected at a density of 1 × 10^6^ in 100 μl PBS into the tail veins of the randomized mice (*n* = 7 per group). The mice were sacrificed after 9 weeks, and the lungs were removed, visually examined, weighed, fixed, and stained with hematoxylin and eosin.

### Statistical analysis

All data values are shown as mean + SEM. Groups and among-group comparisons were conducted using the Student’s *t*-test and analysis of variance, respectively. Differences were regarded as statistically significant when the *P*-value was <0.05.

## Electronic supplementary material

supplementary Figure 1, supplementary Figure 2, supplementary Figure 3, supplementary Figure 4, supplementary Figure 5
