# Supplementary material for: Human colorectal cancer-derived mesenchymal stem cells promote colorectal cancer progression through IL-6/JAK2/STAT3 signaling
Source: Cell Death Dis. 2018 Jan 18;9(2):25. doi: 10.1038/s41419-017-0176-3 (PMC5833830; doi:10.1038/s41419-017-0176-3)
Supplement: Supplementary file 1 — supplementary Figure 1, supplementary Figure 2, supplementary Figure 3, supplementary Figure 4, supplementary Figure 5 [file 41419_2017_176_MOESM1_ESM.ppt]

## Slide 1
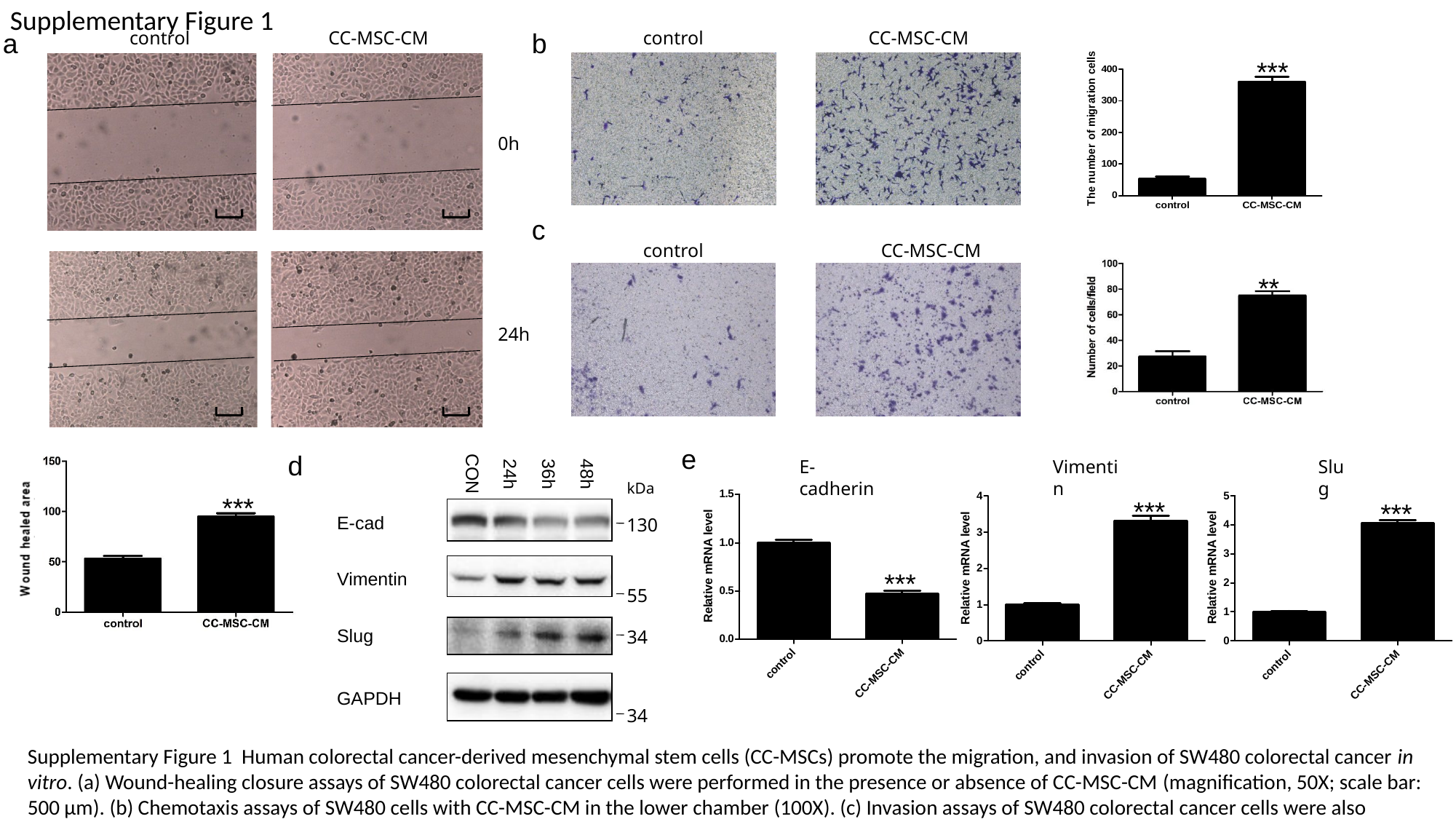

Supplementary Figure 1
a
control
CC-MSC-CM
b
control
CC-MSC-CM
***
0h
c
CC-MSC-CM
control
***
24h
CC-MSC-CM
e
***
d
CON
24h
36h
48h
kDa
E-cad
130
Vimentin
55
Slug
34
GAPDH
34
E-cadherin
Vimentin
Slug
***
***
***
Supplementary Figure 1 Human colorectal cancer-derived mesenchymal stem cells (CC-MSCs) promote the migration, and invasion of SW480 colorectal cancer in vitro. (a) Wound-healing closure assays of SW480 colorectal cancer cells were performed in the presence or absence of CC-MSC-CM (magnification, 50X; scale bar: 500 μm). (b) Chemotaxis assays of SW480 cells with CC-MSC-CM in the lower chamber (100X). (c) Invasion assays of SW480 colorectal cancer cells were also carried out in the presence or absence of CC-MSC-CM (100X). The addition of the CC-MSC-CM enhanced both invasion and migration of SW480 cells. ***P < 0.001 compared with mock treatment. (d) SW620 was cultivated with CC-MSC-CM for different times, and several markers associated with the epithelial-to-mesenchymal transition (EMT) process, such as E-cadherin, vimentin, and SLUG, were detected by western blotting analysis. (e) Real-time polymerase chain reaction (PCR) was carried out using RNA obtained from SW48 cells with and without CC-MSC-CM treatment for 24 h, using primers specific to E-cadherin, Vimentin, Slug, GAPDH, β-actin, and Tubulin in triplicate. Average E-cadherin, Vimentin and Slug RNA levels were normalized to GAPDH, β-actin, and Tubulin.

## Slide 2
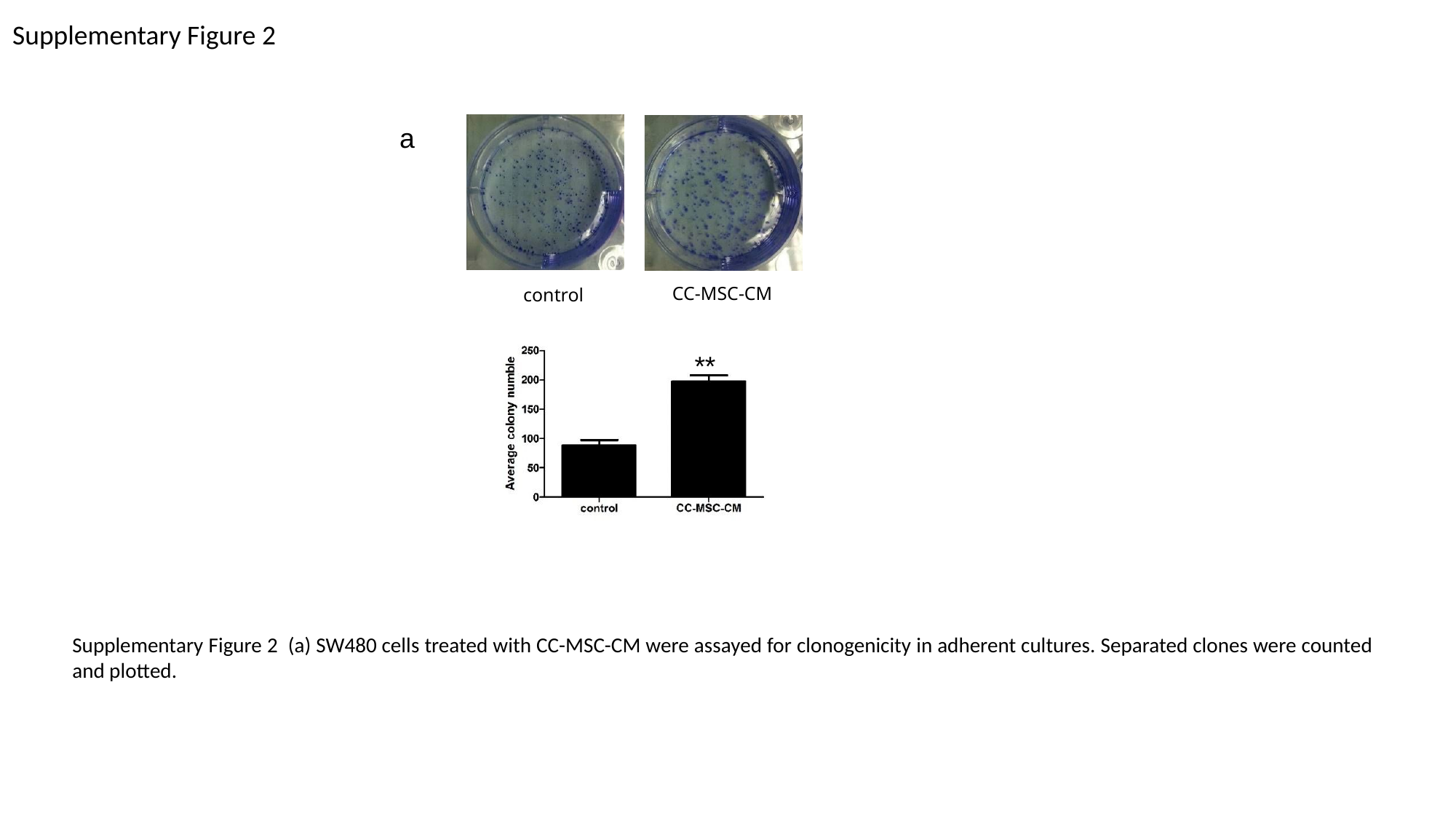

Supplementary Figure 2
a
CC-MSC-CM
control
**
Supplementary Figure 2 (a) SW480 cells treated with CC-MSC-CM were assayed for clonogenicity in adherent cultures. Separated clones were counted and plotted.

## Slide 3
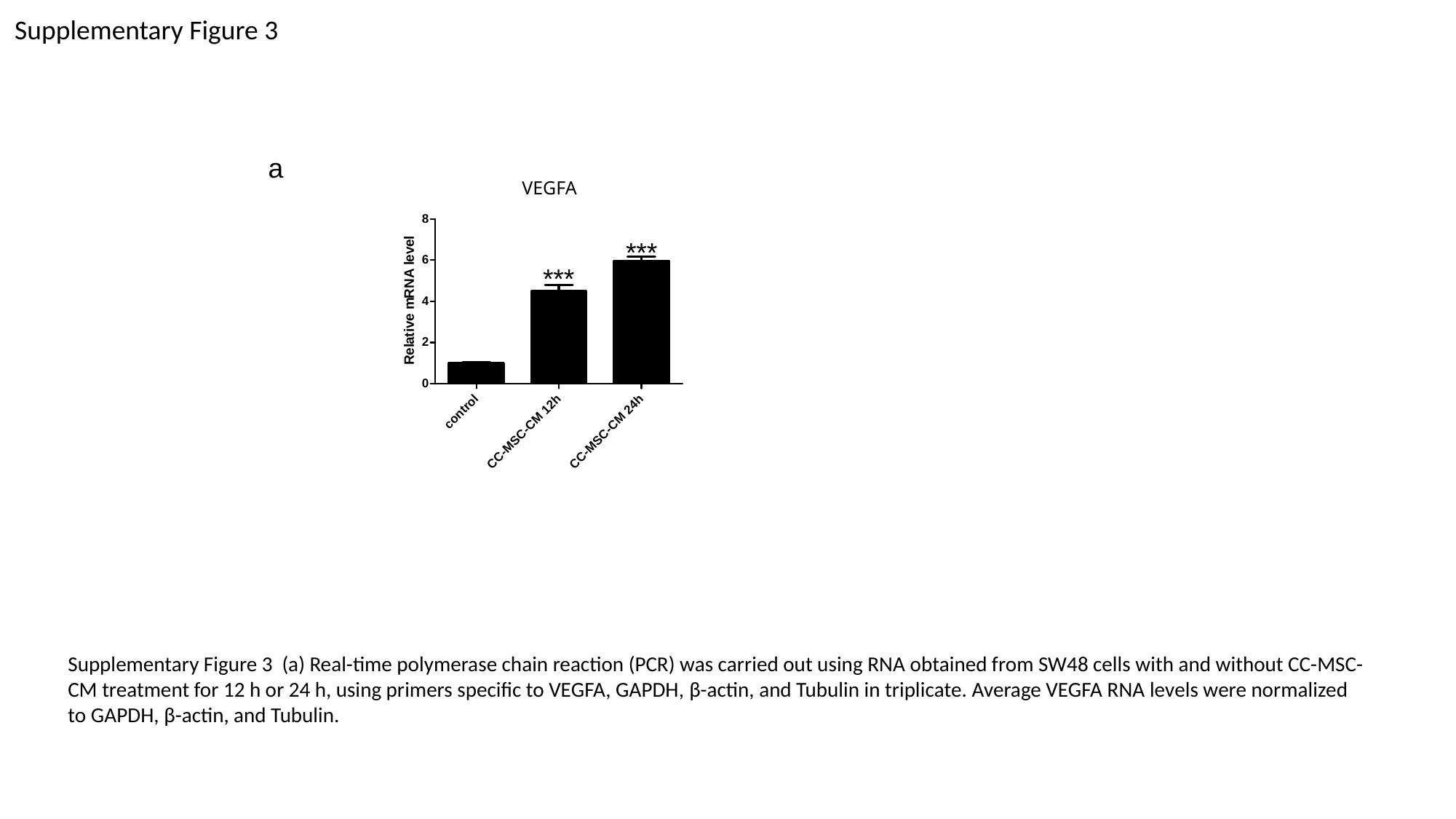

Supplementary Figure 3
a
VEGFA
***
***
Supplementary Figure 3 (a) Real-time polymerase chain reaction (PCR) was carried out using RNA obtained from SW48 cells with and without CC-MSC-CM treatment for 12 h or 24 h, using primers specific to VEGFA, GAPDH, β-actin, and Tubulin in triplicate. Average VEGFA RNA levels were normalized to GAPDH, β-actin, and Tubulin.

## Slide 4
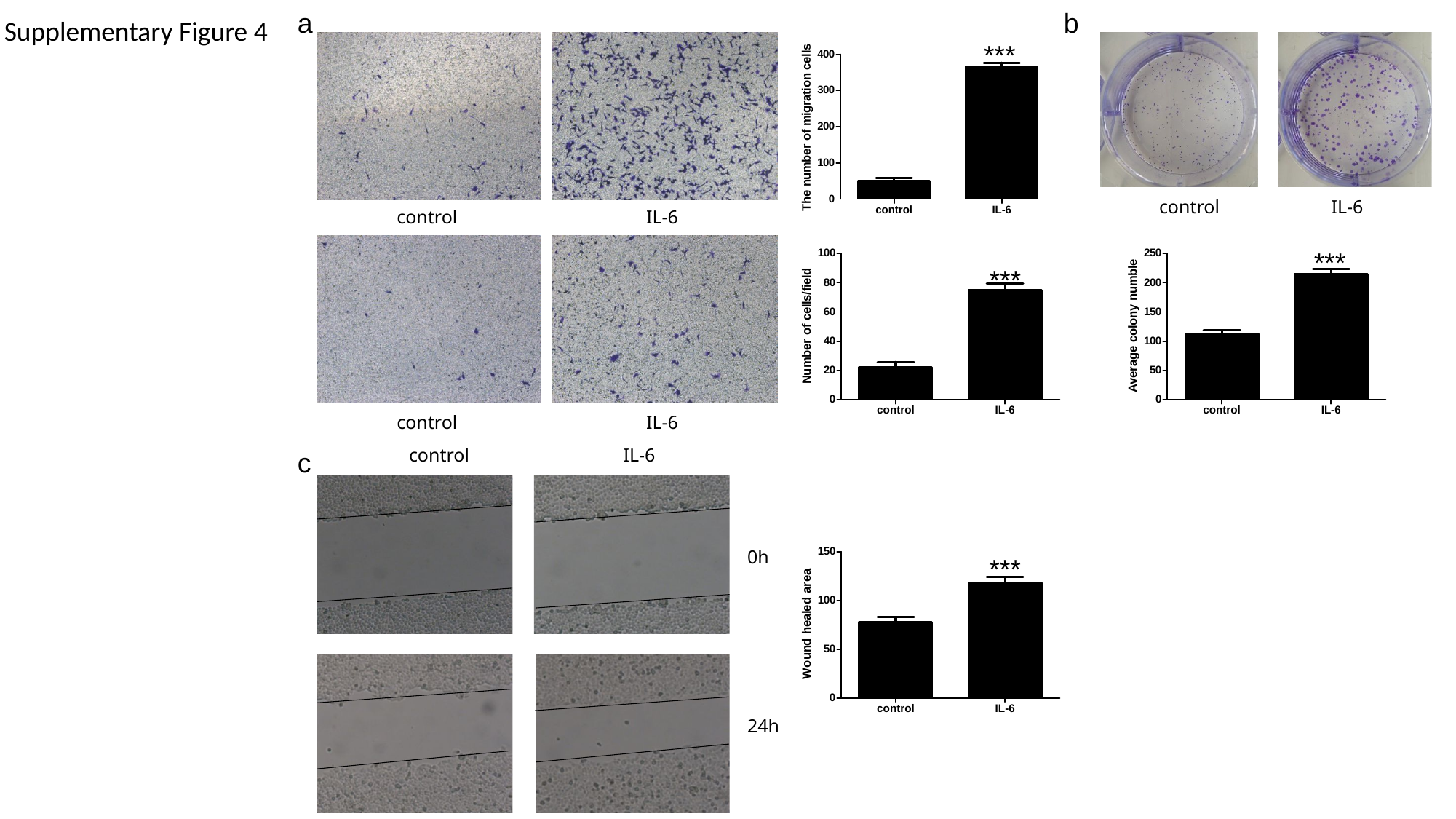

a
b
Supplementary Figure 4
***
control
IL-6
control
IL-6
***
***
control
IL-6
control
IL-6
c
***
0h
24h
Supplementary Figure 4 IL-6 secreted by CC-MSCs enhances the proliferation, migration and invasion of colorectal cancer cells through IL-6/JAK2/STAT3 signaling. (a) Transwell migration (top) and invasion (bottom) assay of SW480 cells with or without 10 ng/mL recombinant IL-6 treatment (100X). (b) Colony-formation assay of SW480 cells treated with or without 10 ng/mL recombinant IL-6. (c) Wound-healing assay of SW480 cells in the presence or absence of recombinant IL-6 (magnification, 50X; scale bar: 500 μm). ***P < 0.001. (d) SW620 cells were cultured with CC-MSC-CM for different times. Protein expression was determined by western blotting and representative results from one of the three independent experiments are presented. (e) SW480 cells were cultured for 30 min in F12 or CC-MSC-CM, which had been pre-incubated with different concentrations of anti-IL-6 antibody for 2 h, and representative results from one of the three independent experiments are presented.

## Slide 5
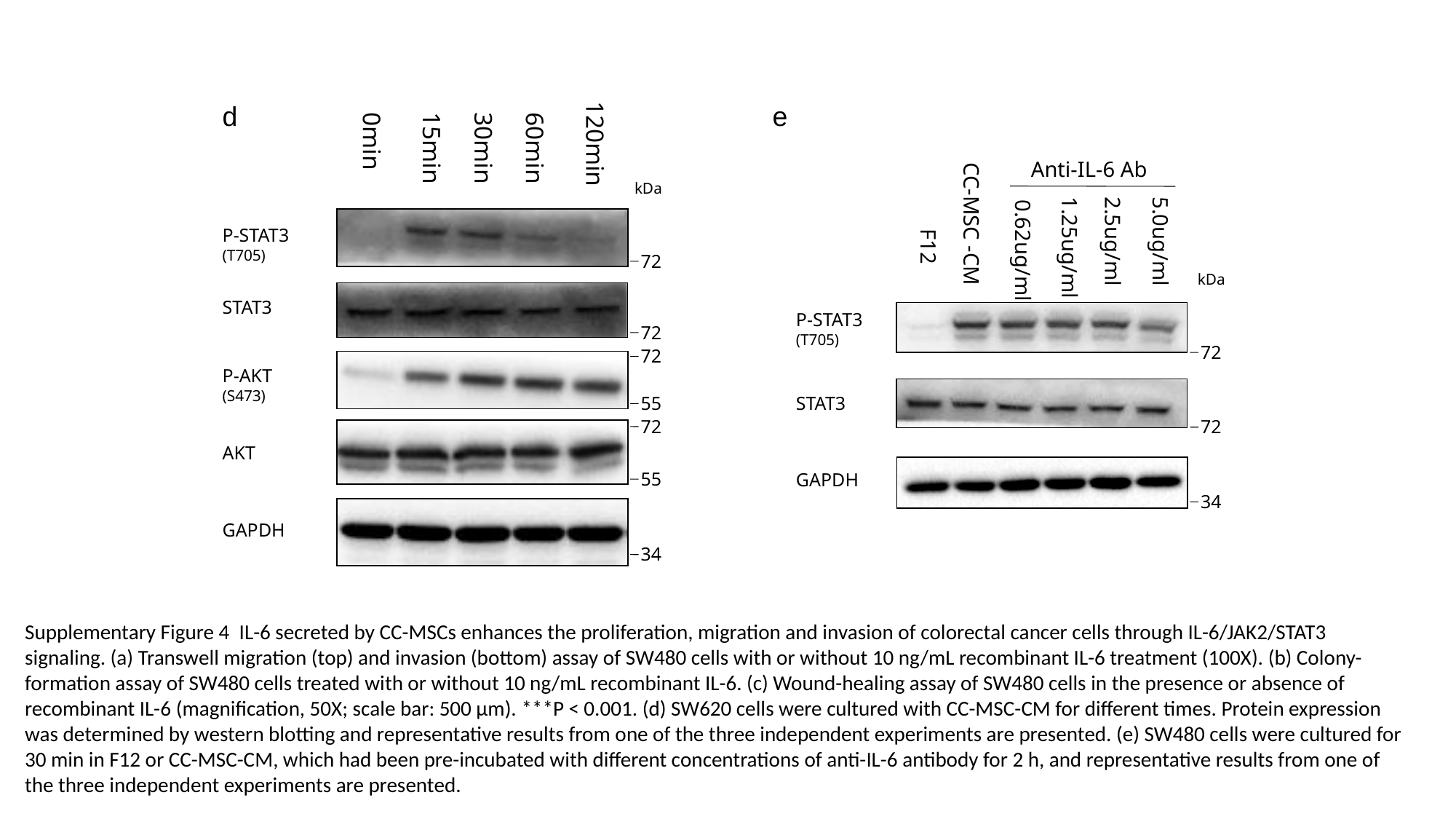

d
e
120min
0min
15min
30min
60min
Anti-IL-6 Ab
CC-MSC -CM
kDa
1.25ug/ml
2.5ug/ml
5.0ug/ml
0.62ug/ml
P-STAT3
(T705)
F12
72
kDa
STAT3
P-STAT3
(T705)
72
72
72
P-AKT
(S473)
55
STAT3
72
72
AKT
55
GAPDH
34
GAPDH
34
Supplementary Figure 4 IL-6 secreted by CC-MSCs enhances the proliferation, migration and invasion of colorectal cancer cells through IL-6/JAK2/STAT3 signaling. (a) Transwell migration (top) and invasion (bottom) assay of SW480 cells with or without 10 ng/mL recombinant IL-6 treatment (100X). (b) Colony-formation assay of SW480 cells treated with or without 10 ng/mL recombinant IL-6. (c) Wound-healing assay of SW480 cells in the presence or absence of recombinant IL-6 (magnification, 50X; scale bar: 500 μm). ***P < 0.001. (d) SW620 cells were cultured with CC-MSC-CM for different times. Protein expression was determined by western blotting and representative results from one of the three independent experiments are presented. (e) SW480 cells were cultured for 30 min in F12 or CC-MSC-CM, which had been pre-incubated with different concentrations of anti-IL-6 antibody for 2 h, and representative results from one of the three independent experiments are presented.

## Slide 6
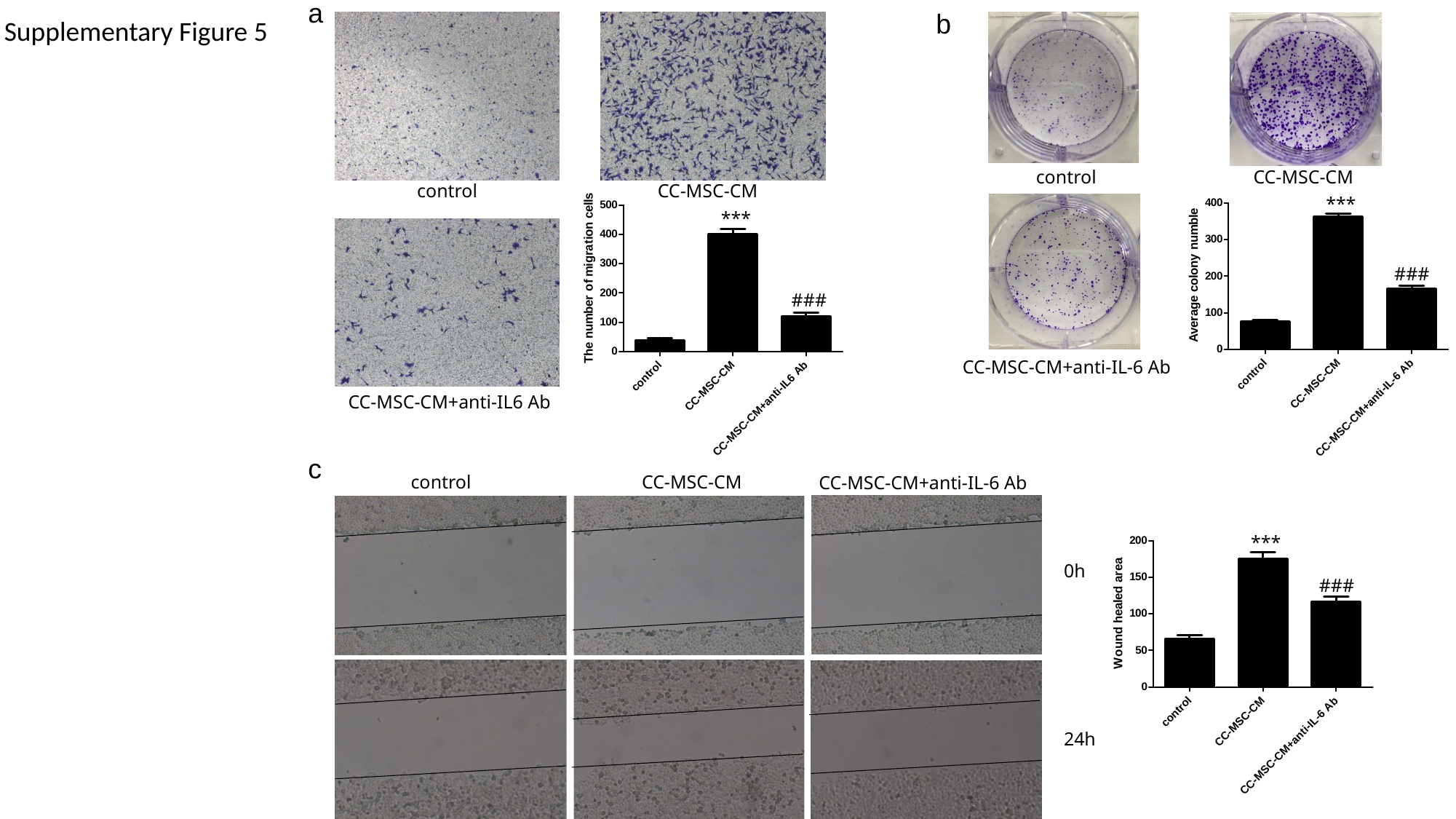

a
b
Supplementary Figure 5
control
 CC-MSC-CM
control
CC-MSC-CM
***
###
***
###
 CC-MSC-CM+anti-IL-6 Ab
CC-MSC-CM+anti-IL6 Ab
c
control
CC-MSC-CM
 CC-MSC-CM+anti-IL-6 Ab
***
0h
###
24h
Supplementary Figure 5 Tumor-promoting effect of CC-MSCs on SW480 cells is reduced by the addition of anti-IL-6 antibody and the inhibitor of STAT3. (a) Transwell migration assay of SW480 cells exposed to CC-MSC-CM with or without anti-IL-6 antibody treatment (100X). (b) Colony-formation assay of SW480 cells treated with CC-MSC-CM in the presence or absence of anti-IL-6 antibody. (c) Wound-healing assay of SW480 cells exposed to CC-MSC-CM was performed in the presence or absence of anti-IL-6 antibody (magnification, 50X; scale bar: 500 μm). (d) Transwell migration assay of SW480 cells exposed to CC-MSC-CM with or without the STAT3 inhibitor (Stattic) treatment (100X). (e) Colony-formation assay of SW480 cells treated with CC-MSC-CM in the presence or absence of the STAT3 inhibitor (Stattic). (f) Wound-healing assay of SW480 cells exposed to CC-MSC-CM was performed in the presence or absence of the STAT3 inhibitor (Stattic) (magnification, 50X; scale bar: 500 μm). *P < 0.05, **P < 0.01, ***P < 0.001: compared with the control group; #P < 0.05, ##P < 0.01, ###P < 0.001: compared with the CC-MSC-CM-treated group.

## Slide 7
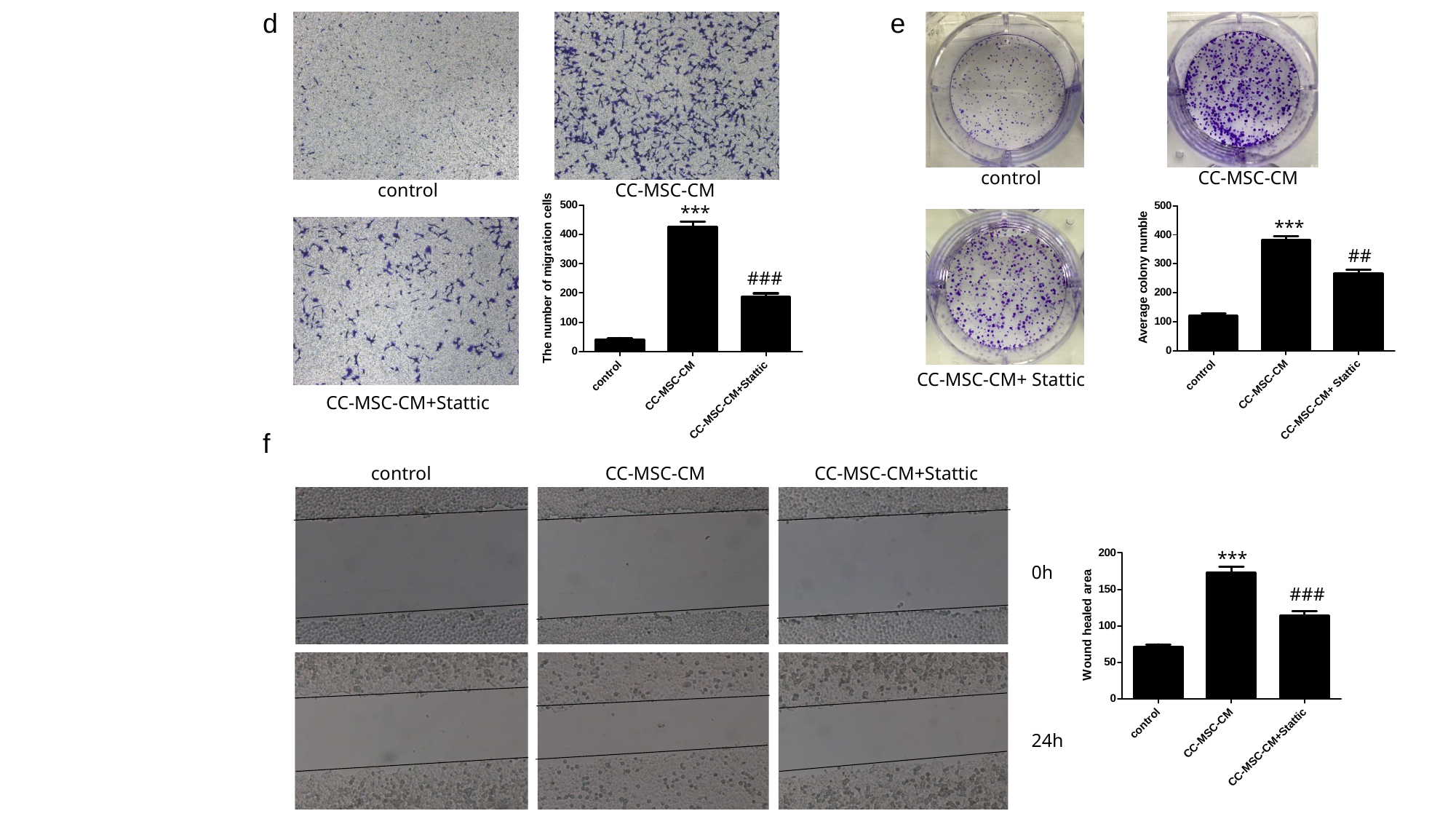

d
e
control
 CC-MSC-CM
control
CC-MSC-CM
***
***
##
###
 CC-MSC-CM+ Stattic
CC-MSC-CM+Stattic
f
control
CC-MSC-CM
CC-MSC-CM+Stattic
***
###
0h
24h
Supplementary Figure 5 Tumor-promoting effect of CC-MSCs on SW480 cells is reduced by the addition of anti-IL-6 antibody and the inhibitor of STAT3. (a) Transwell migration assay of SW480 cells exposed to CC-MSC-CM with or without anti-IL-6 antibody treatment (100X). (b) Colony-formation assay of SW480 cells treated with CC-MSC-CM in the presence or absence of anti-IL-6 antibody. (c) Wound-healing assay of SW480 cells exposed to CC-MSC-CM was performed in the presence or absence of anti-IL-6 antibody (magnification, 50X; scale bar: 500 μm). (d) Transwell migration assay of SW480 cells exposed to CC-MSC-CM with or without the STAT3 inhibitor (Stattic) treatment (100X). (e) Colony-formation assay of SW480 cells treated with CC-MSC-CM in the presence or absence of the STAT3 inhibitor (Stattic). (f) Wound-healing assay of SW480 cells exposed to CC-MSC-CM was performed in the presence or absence of the STAT3 inhibitor (Stattic) (magnification, 50X; scale bar: 500 μm). *P < 0.05, **P < 0.01, ***P < 0.001: compared with the control group; #P < 0.05, ##P < 0.01, ###P < 0.001: compared with the CC-MSC-CM-treated group.
